# Accuracy Improvement of Braking Force via Deceleration Feedback Functions Applied to Braking Systems

**DOI:** 10.3390/s23135975

**Published:** 2023-06-27

**Authors:** Yuzhu Wang, Xiyuan Wen, Hongfang Meng, Xiang Zhang, Ruizhe Li, Roger Serra

**Affiliations:** 1 China Academy of Railway Sciences, Locomotive Car Research Institute, Beijing 100081, China; 2 Intelligent Modelling and Analysis (IMA) Group, University of Nottingham, Nottingham NG7 2RD, UK; 3 INSA Centre Val de Loire, LAMÉ, 41000 Blois, France

**Keywords:** accuracy brake system, dynamic friction coefficient, close-loop braking control, accelerometer application, braking force control

## Abstract

Currently, braking control systems used in regional railways are open-loop systems, such as metro and tramways. Given that the performance of braking can be influenced by issues such as wheel sliding or the properties of the friction components present in brake systems, our study puts forward a novel closed-loop mechanism to autonomously stabilize braking performance. It is able to keep train deceleration close to the target values required by the braking control unit (BCU), especially in terms of the electrical–pneumatic braking transform process. This method fully considers the friction efficiency characteristics of brake pads and encompasses running tests using rolling stock. The test results show that the technique is able to stabilize the actual deceleration at a closer rate to the target deceleration than before and avoid wheel sliding protection (WSP) action, especially during low-speed periods.

## 1. Introduction

**Figure 1:**
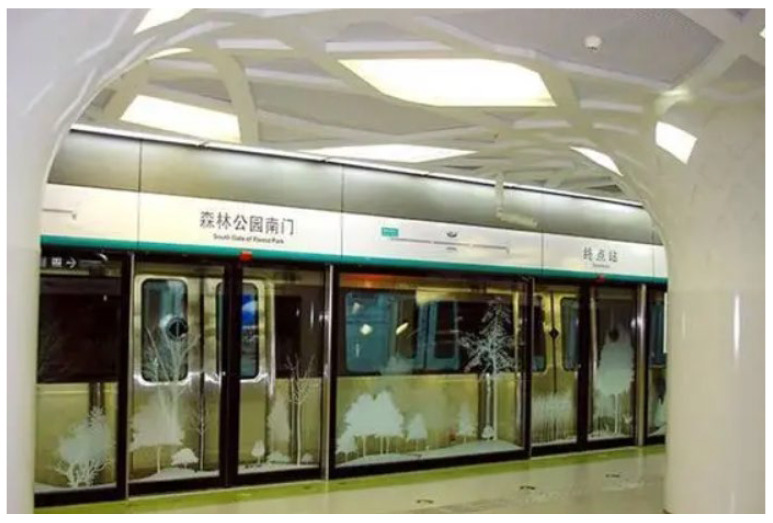
City subway platform, door-to-door arrival.

Urban rail train transit is extensively utilized, and advancements in train braking technology have led to the need for improved control performance in order to enhance the efficiency of urban rail train braking systems. Ensuring accurate parking during operations entails fulfilling more stringent criteria for braking distances and precision. This task becomes especially demanding during peak operation periods, characterized by limited departure intervals and a high volume of passengers. Insufficient control precision in braking directly affects train deceleration and position control accuracy, thereby reducing the overall door-to-door precision throughout the braking process. The graphical representation of this requirement is depicted in Figure 1.

Currently, urban rail train vehicles employ two primary braking methods: electric braking and pneumatic braking. Electric braking is accomplished via the traction system of the train regulating the traction motor to produce reverse torque. In contrast, pneumatic braking employs compressed air, which is transformed into friction via the braking actuator. In the braking procedure, especially during rapid and emergency braking, the objective is to sustain a uniform braking pressure among all wheel sets, taking into account the load conditions of each unit. In theory, this uniform pressure ensures a constant deceleration since the applied braking force remains constant. However, real-world operating conditions prevent the vehicle from achieving ideal circumstances. Even with sufficient adhesion, the actual deceleration deviates from the theoretical value primarily due to variations in the friction coefficient between the brake pad and brake disc friction pair within the brake unit. The variation in the friction coefficient is primarily influenced by the temperature fluctuations experienced by the brake disc before the onset of braking. The energy input, responsible for determining the temperature of the brake disc, is influenced by factors such as the vehicle’s initial braking speed, as well as external conditions, including temperature and humidity. Additionally, running on inclines, curves, tunnels, and other sections of the track generates additional running resistance. The uncertainty associated with the friction braking torque and running resistance contributes to the uncertainty in the overall braking force of the train.

In railway braking systems, the currently used open-loop control system lacks automatic correction based on output during the braking process. Within this system, the brake command functions as the input, whereas the change in train speed serves as the output. Consequently, the accuracy of deceleration control in the braking system is inadequate. Researchers have started investigating closed-loop control techniques for the train braking process to tackle this issue. In the closed-loop control mode, the braking control system employs the braking deceleration as feedback. It adjusts the braking force based on the difference between the target deceleration and the actual deceleration. This approach is known as deceleration closed-loop control.

In 2002, Nankyo proposed the feedback control of deceleration which involved adjusting the braking force of the entire train through the train information management system (TIMS) [1,2]. Closed-loop control using the Smith method was designed to correct the deviation in deceleration between the actual and target values. The feedback control resolved the issue of the actual deceleration, gradually increasing during the braking stage, while the PI controller addressed fluctuation in the early braking period after applying feedback control. Ultimately, the desired braking force of the train was adjusted, resulting in the enhanced precision of the actual deceleration. This study did not specifically examine the causes of deceleration deviation in trains but instead refined the train value theorem by considering the error. However, excessive instructions could potentially induce oscillation in the vehicle’s deceleration. Therefore, other researchers aimed to analyze the load deviation, friction coefficient, rampway, and other parameters during train braking. They proposed the modification of the target braking force through multiple parameter models. Seiji’s research confirmed through simulation that the fuzzy control system exhibited superior parking accuracy and notch change frequency compared to PID control [3]. Ma proposed a parameter predictor for the online estimation of equivalent total disturbance to modify the train braking force, enhancing the accuracy of the actual deceleration, and demonstrated that the predictor is able to meet requirements even in cases of sliding and extreme adhesion [4,5]. In his study, Shin-ichi proposed a distance-based deceleration control method that optimizes braking distance, even when deceleration decays [6]. However, this method does not address the influence of deceleration fluctuations on the braking process and is also limited by wheel adhesion. Nankyo’s study brought attention to the existence of a dead zone within the braking system, which encompasses the response time of the braking control unit (BCU) to commands and the response time of pneumatic components to the BCU. When considering the dynamic adjustment of the closed-loop control, it is crucial to consider the hysteresis between multiple instructions given to the BCU. Consequently, the response time of the braking system becomes a crucial boundary condition. The signal collection, corrected calculation, and execution time of pneumatic components should be minimized to optimize the overall braking process.

To summarize, the main challenge lies in achieving precise control with a multi-parameter controller under limited conditions, particularly when dealing with hysteresis control involving the friction coefficient of the brake disc and the adhesion coefficient of the wheel/rail. In order to tackle this issue, a simplified control system is proposed, which employs a dynamic parameter set of disc coefficients. By effectively handling the complex and variable uncertainties, the system is able to primarily concentrate on the feedback control of the adhesion coefficient between the wheel and rail. This significantly reduces the computational requirements of the system.

The implementation of feedback control for deceleration involves addressing two key issues. First, it requires obtaining accurate real-time information on the train’s deceleration. Second, a suitable brake cylinder pressure correction factor must be determined. Furthermore, the system needs to consider coordinating conditions such as actuator response time and compatibility with other control links within the train to achieve an advanced solution. This involves employing a series of acceleration sensors to accurately measure the overall train deceleration, especially the difference between the front and rear cars during braking and the impact of rampways on deceleration. Furthermore, a set of brake cylinder pressure-modified factors, derived from the characteristics of the brake pads, is employed. It is apparent that the main source of deceleration instability lies in the properties of the brake pads, as their dynamic temperature influences friction efficiency and, consequently, deceleration stability. To tackle this problem, a closed-loop control system is proposed, which utilizes deceleration feedback to stabilize braking performance. This approach functions as a tracking control mechanism. Once the braking demand is determined, the controller selects the appropriate braking deceleration based on the current train speed and allocates it to electric and pneumatic braking. The distribution of braking force is adjusted in real-time if the braking efficiency of the brake pads fluctuates. This technology modifies the pneumatic brake based on the dynamic friction efficiency characteristics of the brake pads. In essence, this technology relies on deceleration feedback to correct the braking effect (deceleration or brake cylinder pressure), aiming to achieve more accurate train stopping and prevent discomfort resulting from deceleration oscillation.

Section 2 presents the framework of the improved deceleration closed-loop control system by comparing it with existing open-loop brake control systems. In Section 3, the utilization of the deceleration sensor and the identification of rail ramps are discussed. The acquisition and utilization of friction coefficient sets are outlined in Section 4. Furthermore, Section 5 presents the design of the deceleration closed-loop control system model, which is based on accurate deceleration feedback information and the friction coefficient set. The functionality of the deceleration closed-loop control system is verified through testing on a rail vehicle in Section 6. Section 7 provides a discussion on potential issues and current bottlenecks in the system at this stage.

## 2. Braking Principle of Urban Rail Trains

In general, the braking control unit oversees the overall braking operation based on the braking command issued by the driver or the automatic train operation system (ATO). This includes managing the distribution and application of braking forces. Two types of braking forces are employed: the electric braking force and the pneumatic braking force. The electric braking force is generated by the traction system and is applied solely to the motor car. The remaining braking force requirements are fulfilled by the pneumatic braking force, which ensures a balanced distribution of braking forces across the entire train, with each unit receiving an appropriate share. The prevailing braking force management mode used in modern urban rail trains is typically marshalling control, which operates at the train level and adheres to the principle of lagging friction braking. In this mode, the electric braking force of motor cars is prioritized, with friction braking utilized as a supplement when the electric braking force is insufficient. As a result, the allocation of braking forces among vehicles is uneven when operating in the multiple-vehicle control mode. Currently, urban rail trains employ electric multiple units (EMU), consisting of two interconnected units, often joined together using close-coupled semi-automatic couplers.

**Figure 2:**
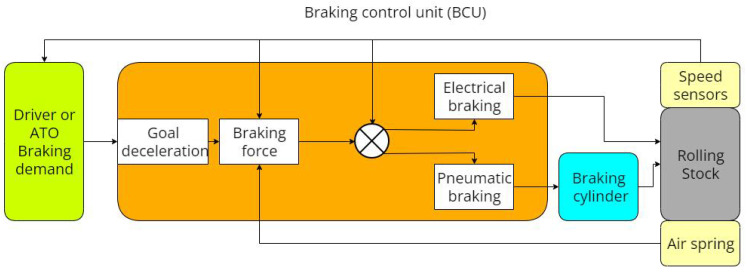
Structure of general brake control system in current railways.

The characteristics of electric braking force result in its minimal contribution at low speeds, with the entirety of braking force being primarily provided by the pneumatic braking force during such instances. During emergency braking scenarios, the pneumatic braking force becomes solely responsible for deceleration. Brake commands typically encompass seven levels, each corresponding to distinct deceleration targets. However, existing brake control systems are structured as open-loop systems, with the brake command serving as the input and train deceleration as the output. Nonetheless, these current brake control systems, as seen in Figure 2, lack the capability to verify whether the train’s deceleration aligns with the desired target.

**Figure 3:**
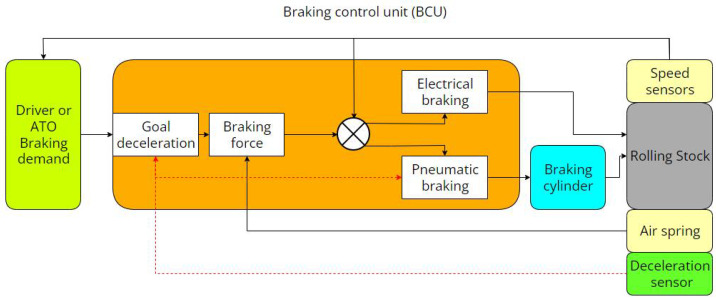
Deceleration feedback control with the deceleration sensor.

The BCU has undergone improvements to achieve and sustain target deceleration until the completion of the braking process, as depicted in Figure 3. Real-time deceleration is monitored using a deceleration sensor, which offers distinct advantages over indirectly obtained vehicle deceleration data from speed sensors. The BCU computes the difference between the actual deceleration and the desired deceleration in real-time and decides whether to activate the modified link. When the criteria are satisfied, the pneumatic braking unit makes necessary adjustments to ensure that the train’s deceleration stays within an acceptable range and maintains stability throughout the entire braking procedure. The selection of modified parameters is based on the time elapsed since the initiation of braking and the current speed. The whole feedback and control loop is shown in the red arrow section.

## 3. Real-Time Deceleration Monitoring

One significant advantage of the new system structure is the incorporation of deceleration sensors, also known as a MEMS-based inertial sensor. It has a limited range but offers high accuracy and resolution. It can achieve highly precise measurements, up to 0.1 m/s^2^, which is essential for accurate control. Unlike the calculation of deceleration based on speed, the use of deceleration sensors eliminates the need for additional calculations, thereby saving data processing time. Furthermore, the deceleration data acquired from these sensors can directly assist in determining the vehicle’s entry and exit points when encountering rampways.

The controller equipped with the deceleration sensor is installed on the train, but the operational vibrations introduced noise into the deceleration signal. To address this, a Kalman filter was employed for filtering. The discrete variable form of the filter offers a simple structure, requires minimal calculations, and facilitates real-time operation in the train controller. It is crucial to address the issue of the deceleration sensor’s sensitivity, as it is able to detect speed fluctuations even during non-braking situations.

During motion, the deceleration sensor is sensitive not only to external acceleration but also to the force of the gravity that it measures. Consequently, the signal from the deceleration sensor represents the combined effect of gravity and external acceleration. Hence, a challenge arises in mitigating the impact of gravitational acceleration during ramp sections. Two potential approaches are discussed in this context. One approach involves treating the gravity component as a measurement error and directly filtering it out during the design of the Kalman filter. The other approach involves determining the train’s state and treating straight sections and rampways separately. The choice between these methods depends on the specific conditions of the railway line. The first method is more suitable for occasional ramps that are not lengthy, as it simplifies the decision-making process for the entire system. However, the second option must be chosen if there is a prolonged ramp state or even parking on a ramp. This is because ramp information will persist for an extended period during the braking decision-making process, and the influence of gravity cannot be disregarded.

### 3.1. Rampway Detector

A crucial component of orientation estimation using an inertial sensor is the determination of external acceleration. This is achieved by subtracting the gravitational acceleration from the deceleration sensor signal. It is important to highlight that this subtraction only necessitates attitude information and does not require the complete 3D orientation [7,8,9]. The relationship between these can be elucidated via the following Equation (1), where $\theta_{ramp}$ represents the angle of the rampway.

(1) $$a_{measure}=a_{actual}+g\sin\theta_{ramp}$$

**Figure 4:**
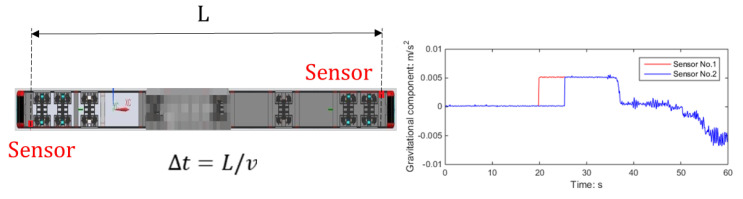
Determination of train attitude based on gravity component.

In railway design standards, the slope of a rampway typically ranges from 20‰ to 30‰. When the ramp is sufficiently long, the train’s attitude can be determined by observing the effect of gravity on the acceleration of the front and rear cars, as shown in Figure 4. In general, these two acceleration signals should have equal magnitudes but opposite directions. However, due to the successive influence of gravity on the acceleration signals within specific time intervals, the train experiences a change in attitude. By considering the train’s speed, these time intervals can be determined. The polarity of the gravity effect can indicate whether the train is moving uphill or downhill.

Theoretically, railway tracks do not have continuous ramps; instead, they resemble the presence of steps, especially when the ramp length is shorter than the train’s length. In such operating conditions, the gravity component is treated as noise and is processed by the filter.

### 3.2. Deceleration Signal Processing

The well-known Kalman filter is one of the fundamental mathematical tools employed for stochastic estimation from noisy sensor measurements. The Kalman filter is a mathematical tool that employs a series of equations to implement a predictor–corrector estimation approach, which is considered optimal under specific assumptions. Its objective is to minimize the error covariance of the estimate. Since its inception, the Kalman filter has found widespread application in the field of navigation, particularly in sensor attitude recognition and precise positioning.

However, when dealing with dynamic systems, time domain signals, such as train acceleration signals, often exhibit nonlinearity. The basic Kalman filter can introduce errors during its operation. In the course of its development, alternatives like the fuzzy adaptive Kalman filter and the nonlinear extended Kalman filter (EKF) have been considered. Ultimately, the nonlinear EKF was chosen due to its computational process and accuracy.

To approximate the estimation around the current estimate, a technique resembling a Taylor series expansion is utilized. For this purpose, partial derivatives obtained from the process and measurement functions are commonly employed.

It is important to note that the distributions of the different random variables no longer adhere to Gaussian distributions once they undergo nonlinear transformations. This demonstrates a fundamental limitation of the EKF. However, through linearization, the EKF can still approximate the optimality of Bayes’ rule [10,11].

The dynamic process is currently governed by a nonlinear stochastic difference equation, as shown in Equation (2):

(2) $$x_{k}=f\left(x_{k-1},u_{k},w_{k-1}\right)$$

With a measurement $z_{k}\in K_{m}$:

(3) $$z_{k}=h\left(x_{k},v_{k}\right)$$

where the random variables $w_{k}$ and $v_{k}$ represent the process and measurement noise. The nonlinear function f in the difference equation relates the state at the previous time step k−1 to the state at the current time step k. This includes driving function $u_{k}$ and the zero-mean process noise $w_{k}$. The nonlinear function h in the measurement Equation (3) relates to the state $x_{k}$ to the measurement $z_{k}$.

One cannot know the individual values of the noise $w_{k}$ and $v_{k}$ at each time step. Nevertheless, it is possible to approximate the state and measurement vectors without explicitly incorporating them into the estimation process.

(4) $${\tilde{x}}_{k}=f\left({\hat{x}}_{k-1},u_{k},0\right)$$

and

(5) $${\tilde{z}}_{k}=h\left({\tilde{x}}_{k},0\right)$$

where ${\tilde{x}}_{k}$ is some a posteriori estimate of the state (from a previous time step) and ${\tilde{z}}_{k}$ is the measurement of the state.

To estimate the process of Equations (6) and (7), which exhibit nonlinear relationships between differences and measurements, additional governing equations are introduced to linearize the estimation of Equations (4) and (5).

(6) $$x_{k}\approx {\tilde{x}}_{k}+A\left(x_{k-1},{\hat{x}}_{k-1}\right)+Ww_{k-1}$$

(7) $$z_{k}\approx {\tilde{z}}_{k}+H\left(x_{k}-{\hat{x}}_{k}\right)+Vv_{k}$$

where

$x_{k}$ and $z_{k}$ denote the actual state and measurement vectors;
${\tilde{x}}_{k}$ and ${\tilde{z}}_{k}$ are the approximate state and measurement vectors from Equations (4) and (5);
${\hat{x}}_{k}$ is a posteriori estimate of the state at step k;
the random variables $w_{k}$ represents the process noise as Q in the basic Kalman filter;
and $v_{k}$ represents the noise of measurement as R in the basic Kalman filter.

A is the Jacobian matrix of partial derivatives of f concerning x, that is

(8) $$A_{\left[i,j\right]}=\frac{\partial f_{\left[i\right]}}{\partial x_{\left[j\right]}}\left({\hat{x}}_{k-1},u_{k},0\right)$$

W represents the Jacobian matrix of partial derivatives of f concerning w,

(9) $$W_{\left[i,j\right]}=\frac{\partial f_{\left[i\right]}}{\partial w_{\left[j\right]}}\left({\hat{x}}_{k-1},u_{k},0\right)$$

H is the Jacobian matrix of partial derivatives of h concerning x,

(10) $$H_{\left[i,j\right]}=\frac{\partial h_{\left[i\right]}}{\partial x_{\left[j\right]}}\left({\tilde{x}}_{k},0\right)$$

V is the Jacobian matrix of partial derivatives of h concerning v,

(11) $$V_{\left[i,j\right]}=\frac{\partial h_{\left[i\right]}}{\partial v_{\left[j\right]}}\left({\tilde{x}}_{k},0\right)$$

It should be noted that, for simplicity in the notation, the time step subscript k is not used with the Jacobians A*,* W*,* H*,* and V, although they are different at each time step.

Now a new notation for the prediction error can be defined as

(12) $${\tilde{e}}_{x_{k}}\equiv x_{k}-{\tilde{x}}_{k}$$

and the measurement residual as

(13) $${\tilde{e}}_{z_{k}}\equiv z_{k}-{\tilde{z}}_{k}$$

Since $x_{k}$ and $z_{k}$ are actual existing vectors, we can rewrite Equations (12) and (13) with an error process as

(14) $${\tilde{e}}_{x_{k}}\approx A\left(x_{k-1}-{\tilde{x}}_{k-1}\right)+\varepsilon_{k}$$

(15) $${\tilde{e}}_{z_{k}}\approx H{\tilde{e}}_{x_{k}}+\eta_{k}$$

where $\varepsilon_{k}$ and $\eta_{k}$ represent new independent random variables having zero mean and covariance matrices $WQW^{T}$ and $VRV^{T}$, with Q as representing the process and R as measurement noise.

Then, the estimate of Equation (14) called ${\hat{e}}_{k}$ can be used, along with Equation (12), to obtain the a posteriori state estimates for the original nonlinear process as

(16) $${\hat{x}}_{k}={\tilde{x}}_{k}+{\hat{e}}_{k}$$

Given these approximations and letting the predicted value of ${\hat{e}}_{k}$ be zero, the Kalman filter equation used to estimate ${\hat{e}}_{k}$ can be given as

(17) $${\hat{e}}_{k}=K_{k}{\tilde{e}}_{z_{k}}$$

By substituting Equation (17) back into Equation (16) and making use of Equation (13),

(18) $$z_{k}={\tilde{x}}_{k}+K_{k}\left(z_{k}-{\tilde{z}}_{k}\right)$$

Now, Equation (18) can be used for the measurement update in the extended Kalman filter, with ${\tilde{x}}_{k}$ and ${\tilde{z}}_{k}$ that are taken from Equations (4) and (5). The Kalman gains $K_{k}$ as the basic Kalman filter with the appropriate substitution for the measurement error covariance.

The subscript k is applied to the Jacobians A, W, H, and V to highlight their features that are different at each time step and need to be recomputed. Therefore, the time update equations for the EKF are as follows:

(19) $${\hat{x}}_{k}^{-}=f\left({\hat{x}}_{k-1},u_{k},0\right)$$

(20) $$P_{k}^{-}=A_{k}P_{k-1}A_{k}^{T}+W_{k}Q_{k-1}W_{k}^{T}$$

The time update equation enables the projection of state and covariance estimates from the previous time step (*k* − 1) to the current time step (k).

Again f in Equation (19) comes from Equation (4), $A_{k}$ and $W_{k}$ are the processes Jacobians at step k, and $Q_{k}$ is the process noise covariance at step k, as the basic Kalman filter. The EKF time measurement equations are as follows:

(21) $$K_{k}=P_{k}^{-}H_{k}^{T}{\left(H_{k}P_{k}^{-}H_{k}^{T}+V_{k}R_{k}V_{k}^{T}\right)}^{-1}$$

(22) $${\hat{x}}_{k}={\hat{x}}_{k}^{-}+K_{k}\left(z_{k}-h\left({\hat{x}}_{k}^{-},0\right)\right)$$

(23) $$P_{k}=\left(1-K_{k}H_{k}\right)P_{k}^{-}$$

**Figure 5:**
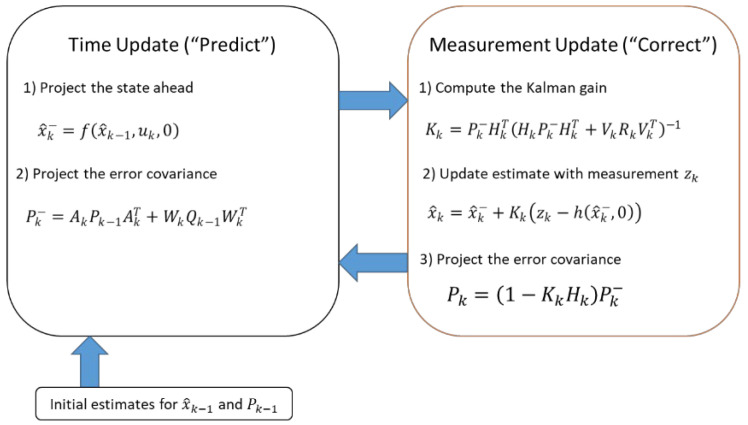
Operation of the extended Kalman filter.

Again, h in Equation (22) comes from Equation (5), $H_{k}$ and V are the measurement Jacobians at step k, and $R_{k}$ is the measurement noise covariance at step k. The functioning of the EKF is comparable to that of the standard Kalman filter, as depicted in Figure 5.

To assess the acquisition accuracy of the deceleration sensors, the acceleration information derived from the differentiation of the speed sensor will be utilized for comparison. This allows for fault judgments and analyses to be conducted.

## 4. Variable Friction Coefficient

Traditionally, a braking control system assumes a constant friction coefficient between the brake disc and the pad, simplifying the analysis. This assumption relies on the understanding that variations in the friction coefficient have a negligible impact. However, the friction coefficient of disc brake pads can undergo significant changes during braking, primarily due to factors like temperature. Consequently, the coefficient of friction can significantly influence the braking system’s performance. To enhance braking accuracy and accurately calculate the final braking force, it becomes imperative to consider the effect of the variable coefficient of friction. This consideration is essential for improving braking accuracy [12,13].

### 4.1. Friction Coefficient Model

**Figure 6:**
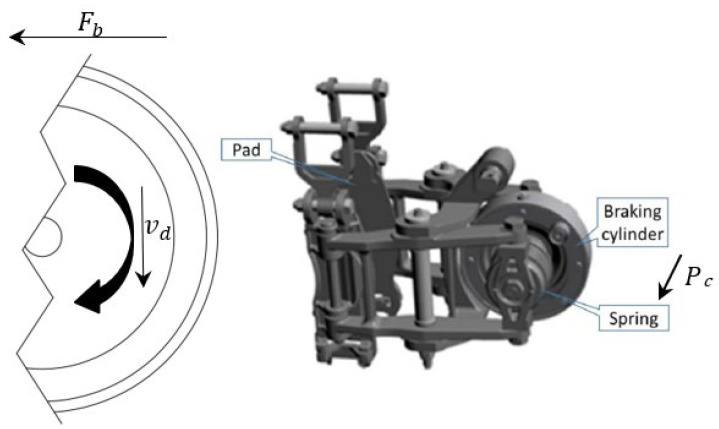
The mechanical structure of the brake clamp unit.

The fundamental principle of the brake actuation system in metro trains involves converting the vehicle’s kinetic energy into thermal energy through friction. Mechanical braking, as opposed to electric braking, is a traditional and dependable method for train braking. Tread braking and disc braking are the two common forms of braking, with this study primarily focusing on the wheel-disc configuration. The brake actuation unit commonly comprises a brake pad, a wheel-mounted disc brake, and the corresponding drive components. The relative motion between the brake pad and the disc brake produces the frictional force, which is regulated by the brake cylinder. Figure 6 illustrates an example of a wheel-mounted disc and a brake caliper equipped with brake pads.

The braking force exerted by the braking device corresponds to the frictional force generated upon contact between the brake pad and the brake disc. Usually, the air pressure $P_{c}$ provided by the brake cylinder acts upon the piston of the brake cylinder, resulting in the piston rod to generate thrust, which is amplified by the lever system of the basic brake device and finally converted into brake pad pressure and applied to the brake disc. Equation (24) is the braking force $F_{b}$ in regard to the braking time.

(24) $$F_{b}\left(t\right)=P_{c}(t)\cdot \theta \cdot \mu_{d}(t)$$

where θ is the structure parameter determined by the design of the mechanism, which considers the lever ratio of the calliper, the radius of the effective braking force, and the number of wheel discs, etc. $\mu_{d}$ represents the dynamic variable friction coefficient between the disc and pad. It is apparent that the braking force is influenced by two variables: the brake cylinder pressure and the friction coefficient.

The contact characteristics between the brake disc and the brake pad can be evaluated through dedicated tests, and detailed methodologies for these tests can be found in the references provided [14]. The calculation formula for the braking force in the braking control system employs the average friction coefficient derived from testing. Nonetheless, it is crucial to acknowledge that the instantaneous friction coefficient is a dynamic parameter that undergoes changes over time during braking. The varying nature of the coefficient of friction can impact the braking efficiency, especially during short-term braking scenarios. While the average friction coefficient can be employed for general braking force calculation and distribution, precise control necessitates the consideration of the instantaneous friction coefficient.

The friction coefficient between the brake disc and brake pad is mainly influenced by the temperature and relative speed of the contacting surfaces. It has been observed that the instantaneous friction coefficient undergoes a significant change from the start of the braking process compared to the steady-state condition. Building upon this empirical observation, Equation (25) is introduced to this paper to capture the variation in friction force between the brake disc and brake pad.

(25) $$\mu_{d}\left(t\right)=\mu_{d0}\left(n_{v}e^{-m_{v}v_{d}\left(t\right)}+1\right)\left(n_{T}e^{-m_{T}T_{d}\left(t\right)}+1\right)$$

where $\mu_{d0}$ is the steady-state friction coefficient of the surface between the disc and brake pad, $n_{v}$ is a multiplication factor resulting from the friction speed, $m_{v}$ is a parametric coefficient given by the exponential function of the speed $v_{d}$, and $v_{d}\left(t\right)$ is the relative speed between the contact surface of the disc and the pad during the braking time t, and its value can be calculated based on train speed and wheel information. $n_{T}$ is a multiplication factor due to the temperature increasing, $m_{T}$ is a parametric coefficient by the exponential function of the temperature $T_{d}$, and $T_{d}\left(t\right)$ is the temperature change during the braking time t.

To obtain the disc temperature increase $T_{d}\left(t\right)$ in Equation (25), a dedicated test bench is used to monitor the temperature change process under different speed classes. Through the analysis of the temperature curve of the brake pad, it becomes possible to determine the corresponding friction coefficient for each speed category.

### 4.2. Friction Coefficient Set Based on Long Short-Term Memory

Utilizing existing data as an adjustment factor to correct the brake cylinder pressure presents challenges. To alleviate the computational burden on the BCU, a preset adjustment coefficient set is necessary for the BCU’s calculator. This coefficient set should cover all potential speed categories of the train. To accomplish this, machine learning techniques are employed to forecast the performance of various speed grades. However, the utilization of data prediction methods to supplement untested data presents challenges in this approach, greatly affecting the reliability of the coefficient set. Among the models considered, LSTM is favored for predicting the optimal set of friction coefficients due to its ability to process and forecast important events with long intervals and time delays in time series data. It should be noted that other models, such as moving average, linear regression, AutoARIMA, and prophet, were explored during the method selection process. However, many algorithms encounter challenges in accurately capturing the correlation between the friction coefficient and speed level, especially when it comes to fitting the braking duration.

Long short-term memory (LSTM) is a type of artificial neural network employed in the fields of artificial intelligence and deep learning. Unlike conventional feed-forward neural networks, LSTM incorporates feedback connections [15]. The name LSTM derives from its analogy to a standard recurrent neural network (RNN), which possesses both “long-term memory” and “short-term memory.”

In conventional RNNs, node outputs are exclusively determined by weights, biases, and activation functions. Additionally, each time step utilizes the same parameters within a sequential structure. LSTM introduces a gate mechanism that governs the flow and retention of information, addressing the long-term dependency issue encountered in RNNs [16].

**Figure 7:**
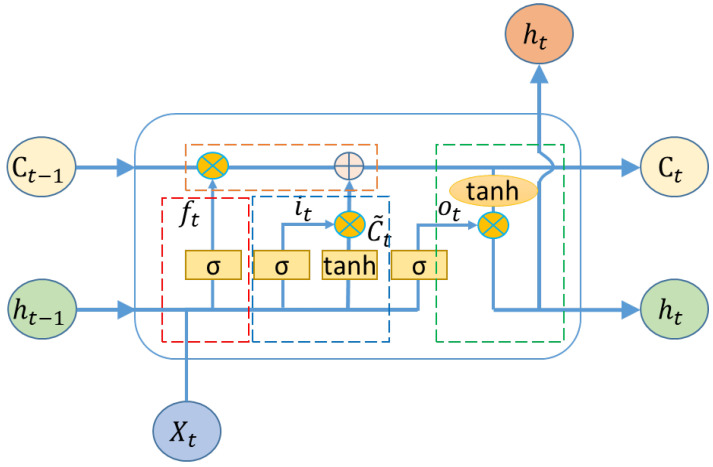
Schematic diagrams of LSTM [16].

Figure 7 illustrates the four components of the LSTM model, namely the forget gate, input gate, output gate, and cell state. Several parameters are mentioned, namely $X_{t}$, the input; $C_{t}$, the cell stat for long-term memory; and $h_{t}$, the hidden layer for short-term memory.

In the initial stage, marked in red, the determination of which information to discard from the cell state is performed by a layer referred to as the forget gate. This gate reads $h_{t-1}$ and $x_{t}$, and outputs a value between 0 and 1 for each digit in the cell state $C_{t-1}$, where 1 means “keep completely” and 0 means “discard completely”.

(26) $$f_{t}=\sigma \left(W_{f}\cdot \left|h_{t-1}-x_{t}\right|+b_{f}\right)$$

The input gate, marked in blue, consists of two parts, $i_{t}$ and ${\tilde{C}}_{t}$. Initially, the input gate layer, which is a sigmoid layer, determines the values that will be updated. Then, a new vector of candidate values is created by a *tanh* layer, where ${\tilde{C}}_{t}$ is a new estimation of the cell state.

(27) $$i_{t}=\sigma \left(W_{i}\cdot \left|h_{t-1}-x_{t}\right|+b_{i}\right)$$

(28) $${\tilde{C}}_{t}=tanh\left(W_{c}\cdot \left|h_{t-1}-x_{t}\right|+b_{f}\right)$$

LSTM generates new states by performing addition operations instead of multiplication. The result $C_{t}$ is stored as a new long-term judgment of the cell state. The addition operation in LSTM effectively resolves the problem of gradient vanishing or exploding, which is a common issue in simple RNNs.

(29) $$C_{t}=f_{t}\times C_{t-1}+i_{t}\times {\tilde{C}}_{t}$$

The output gate layer, marked in green, is calculated through input information $X_{t}$ and the hidden layer of the last step is given as $h_{t-1}$.

(30) $$o_{t}=\sigma \left(W_{o}\cdot \left|h_{t-1}-x_{t}\right|+b_{o}\right)$$

(31) $$h_{t}=o_{t}\times tanh\left(C_{t}\right)$$

By arranging multiple LSTM cells, it becomes possible to process sequential data inputs. These LSTM cells are typically organized in layers, where the output of each cell serves as the input for subsequent cells. The incorporation of a layered structure enhances the capacity of the network and allows for the capture of more complex dependencies within the data.

Having grasped the operating principle of LSTM for data forecasting, the friction coefficient data from existing speed categories are utilized as the training set, with the missing data predicted at 10 km/h intervals. To economize computational resources, the dataset is selectively sampled at suitable time intervals, with the selection of this interval being guided by the response time of the pneumatic components within the braking system.

Therefore, the obtained results μ(v,t) are shown in the figure, and this coefficient set still has room for optimization. This depends on the number of training sets used for data forecasting and the acceptable level of bias.

## 5. Deceleration Feedback Closed-Loop Control

**Figure 8:**
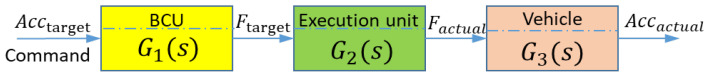
Schematic diagram of the braking system.

As shown in Figure 8, the generalized brake control system discussed earlier can be segmented into three components: the brake control unit, the brake execution unit, and the controlled entity. In these, $G_{1}\left(s\right)$ is the transfer function of the braking control unit. After a braking command is issued, the transfer function assesses the required deceleration based on the braking intensity. It then calculates the target braking force and proceeds to distribute the braking force accordingly. $G_{2}\left(s\right)$ is the transfer function of the braking execution link. The braking force is imposed on the vehicle through the braking apparatus. Nonetheless, due to the impact of uncertain parameters, like the device’s internal friction coefficient, the actual braking force may diverge from the target braking force. $G_{3}\left(s\right)$ is the transfer function of the controlled object, which is mainly affected by the adhesion between the wheel and the rail. Ultimately, the control system yields the actual deceleration.

The target deceleration is determined by the brake control unit based on the braking class and train speed provided by the controller. The brake control unit determines the required brake cylinder pressure $P_{c0}$. The calculation involves a transfer function, denoted as $G_{1}\left(s\right)$, which incorporates the distribution of electro-pneumatic braking force. However, a detailed discussion of this transfer function is beyond the scope of this study.

(32) $$P_{c0}=\frac{F_{b0}}{n\cdot \frac{\pi }{4}D^{2}\cdot \xi \cdot \zeta \cdot \mu_{d0}}=\frac{m_{0}(1+\gamma )\cdot a_{0}}{n\cdot \frac{\pi }{4}D^{2}\cdot \xi \cdot \zeta \cdot \mu_{d0}}$$

where n is the number of brake callipers; D denotes the diameter of the brake cylinder; ξ is the brake magnification; ζ is the mechanical efficiency of the brake calliper; $F_{b0}$ is the target braking force; and $a_{0}$ is the target deceleration. Among these parameters, the majority are device-specific, indicating that they remain constant for a particular device.

Broadly speaking, the brake execution unit is composed of two segments: the pneumatic control group and the fundamental brake group.

The charging and exhausting process of the brake cylinder pressure by the rail vehicle pneumatic brake system can be given in a simplified version as a first-order inertial link and its transfer function $G_{q}\left(s\right)$ is shown in Equation (33)

(33) $$G_{q}\left(s\right)=\frac{1}{1+T_{q}s}$$

In Equation (33), $G_{q}\left(s\right)$ represents the transfer function of the charging and exhausting process of the brake cylinder pressure and $T_{q}$ represents the inertial time constant of the charging and exhausting characteristics of the brake cylinder. Due to the existence of $T_{q}$, the response time largely affects adjustment speed of the brake cylinder pressure, that is, the actual corresponding relationship is that the deceleration corresponding to the brake cylinder pressure $P_{c(t-T_{q})}$ is actually $a_{t}$.

### 5.1. Braking Force Correction Based on the Friction Coefficient Model

Upon comparing Equations (24) and (32), it becomes apparent that the discrepancy between the desired and actual braking force arises from the variance between the average friction coefficient and the actual friction coefficient.

**Figure 9:**
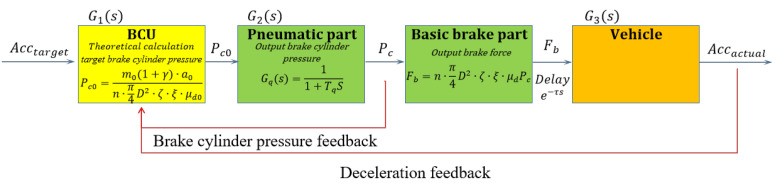
Schematic diagram of the braking system with deceleration feedback.

As depicted in Figure 9, two closed-loop control links can be established. The first loop encompasses the target and actual deceleration, and the second loop comprises the target and actual braking force. However, given the challenge of measuring the actual braking force, brake cylinder pressure and deceleration are utilized as reference values for the closed-loop control.

It is essential to recognize that this system operates as an additional element beyond the standard braking control. There are several reasons for not opting to incorporate this functionality into the core control of the BCU. Firstly, it is imperative to ensure that the fundamental braking function remains unaffected by the computation, thereby guaranteeing the promptness of braking. Secondly, in the case of anti-skid and emergency braking commands, their priority overrides the necessity for precise parking. When conflicting demands arise, the emphasis is placed on braking requirements, which results in the disengagement of the deceleration closed-loop control chain. In essence, the BCU computes the braking demand based on a fixed friction coefficient and then optimizes it through a dynamic friction coefficient. However, the optimization process can be bypassed, a decision taken from a system security viewpoint.

### 5.2. Function Activation Threshold

The initiation of the entire deceleration feedback control module is governed by the ratio of actual to target deceleration. Upon the commencement of braking, should the ratio between the two fail to fall within the range of 90–110% for a duration exceeding 0.5 s, the deceleration closed-loop control will be engaged. The brake cylinder pressure will be adjusted proportionally, and the coefficient φ refers to the quation as follows:

(34) $$\varphi =\frac{a_{actual}}{a_{target}}\approx \frac{P_{c(t-T_{q})}}{P_{c0}}=\frac{\mu_{d}(v,t-T_{q})}{\mu_{d0}}$$

In cases when the deceleration still cannot meet the requirements after correction, then $T_{q}$ needs to be adjusted next time. If the specified conditions are met, the adjustment continues based on the predefined coefficient set until the completion of the braking procedure. Of course, the ideal state of the whole process would be to use $\mu_{d}(v,v_{t})$ as the correction coefficient and to control it completely according to the gradient curve of the expected train speed. However, under unfavorable track conditions, the speed’s decline may not align with the ideal state, thereby hindering the successful alignment with the appropriate friction coefficient.

During the determination of a vehicle’s slope position via the acceleration signal, it is imperative to remove the component of gravity for an accurate assessment of the actual deceleration. This task can generally be accomplished in two ways. When the deceleration signals from the front and rear cars encounter an incline, a temporary disparity is observed. Additionally, while on the slope, a consistent difference will persist between the measured deceleration signal and the deceleration calculated by differentiating the speed signal.

Upon receiving a braking command for either rapid or emergency braking, the deceleration feedback control remains inactive. When the wheel slide protection function is activated, the deceleration closed-loop control module is deactivated. At this point, it becomes necessary to assess the modification factor. If the modification process amplifies the brake cylinder pressure and the deceleration closed-loop control is subsequently released, the state preceding the modification is not restored. In contrast, the adjusted brake cylinder pressure is preserved to prevent an excessive braking force when braking is re-engaged.

### 5.3. Enable and Disable Conditions

Serving as an auxiliary measure, the deceleration closed-loop control system does not impact the primary control functionality of braking from a safety standpoint. Primarily, this control system is designated for the pneumatic aspect of traditional and accelerated braking. While emergency braking is also a facet of pneumatic braking, the maximum braking efficiency is given priority, thus excluding the involvement of the deceleration closed-loop control system. When the wheel slide protection feature is triggered, the deceleration closed-loop control is deactivated and the deceleration is reduced. If the deceleration does not meet the error tolerance after the activation of the deceleration closed-loop control system, the closed-loop control will be disabled.

## 6. Test Verification

A specially designed test train was employed to validate the efficacy of the deceleration closed-loop control system. The total weight of the train simulates the operational conditions under full load. The train operates on a route punctuated by extended slopes. The comprehensive validation procedure comprised the arrangement of the deceleration sensors, a brake disc examination, the projection of the corresponding friction coefficient set, and a track test.

### 6.1. The Deceleration Sensor Arrangement

**Figure 10:**
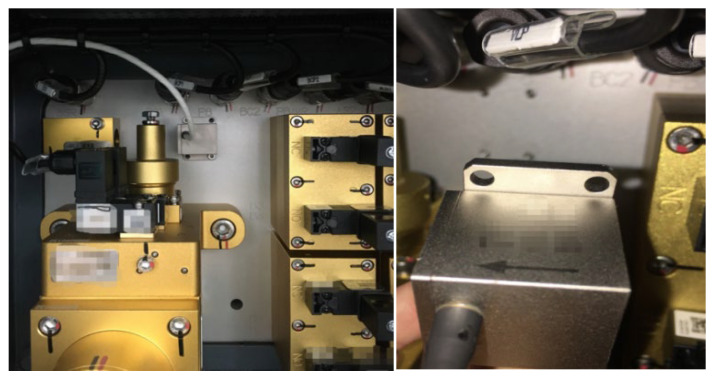
Installation mode of deceleration sensor.

To meet the ramp judgment criteria outlined earlier, two uniaxial deceleration sensors were mounted on the front and rear vehicles, with their respective parameters provided in Table 1. These sensors were positioned inside the brake control device box, as illustrated in Figure 10. It is worth noting that the deceleration sensor is oriented in the positive direction away from the center of the train. During actual operation, the train’s forward direction is determined by the speed signal, and the signal in the opposite direction is appropriately converted.

**Figure 11:**
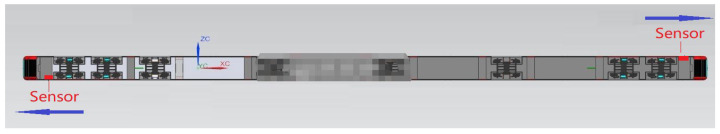
Installation position of deceleration sensor.

The positioning of the deceleration sensor is illustrated in Figure 11. However, it is advisable to install the sensor along the centerline of the train, particularly in cases where the curvature of the track is tight. This is because, during turns, there may be a difference in acceleration between the inner and outer sides, which could be detected by the system and potentially impact its decision-making process.

### 6.2. Brake Disc Test

**Figure 12:**
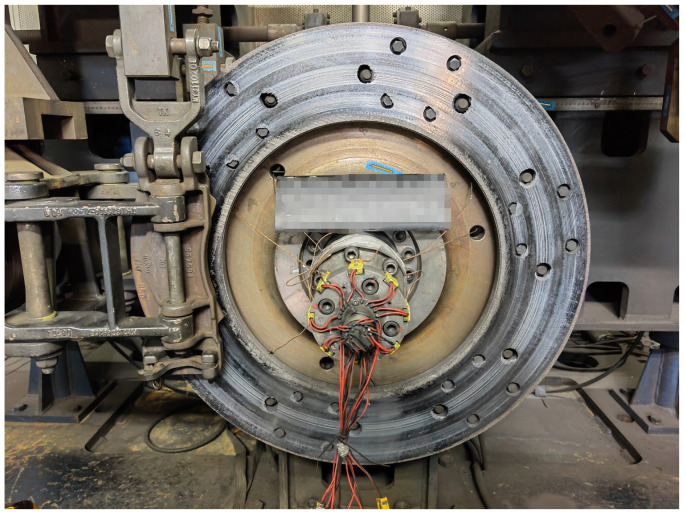
Brake disc performance test.

Collecting the performance data of the brake disc prior to the line test is crucial. To achieve this objective, a specialized testing apparatus is utilized to investigate the evolving pattern of brake disc temperature and friction coefficient during the braking procedure across different speed categories. The results of this test are depicted in Figure 12.

**Figure 13:**
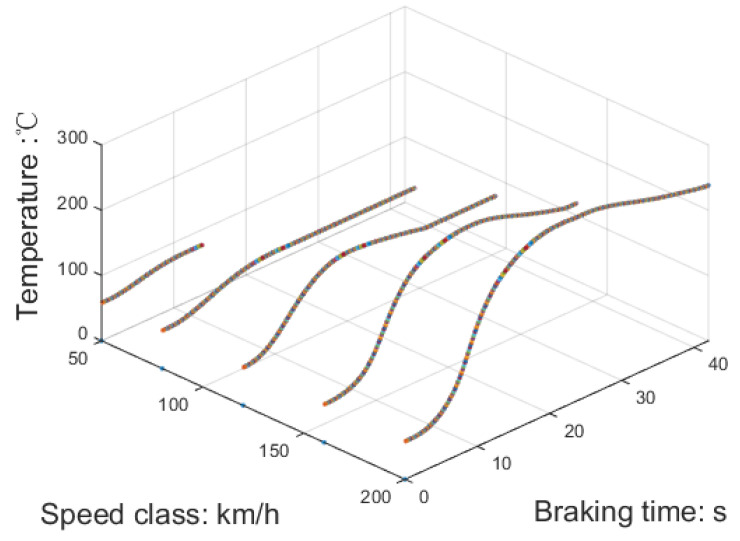
Temperature of the surface with different speed classes and braking time.

Due to the time constraints of the test, only commonly used speed classes, namely 50, 80, 120, 160, and 180 km/h, were selected as the initial speeds for the simulated braking test. Figure 13 presents the temperature curve of the brake pad, serving as the test specimen, throughout the braking test.

**Figure 14:**
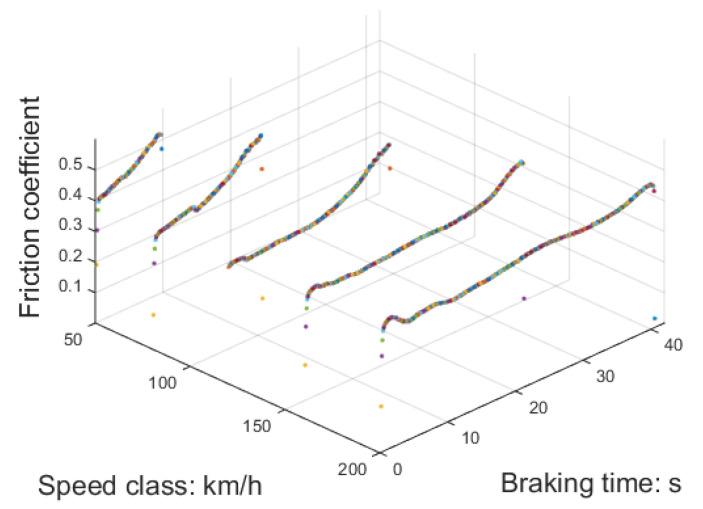
Effectiveness of the different speed classes and braking time.

Using the temperature of the braking disc as a reference, the friction coefficient at different speed classes can be determined and are illustrated in Figure 14. It is evident that there is a substantial rise in the friction coefficient when the initial braking speed is 50 km/h. However, as the initial speed of braking increases, the alteration in the friction coefficient becomes less prominent. To ensure a more consistent braking force, the pressure applied to the brake cylinder should be adjusted to counteract this changing trend.

### 6.3. Prediction of the Corresponding Friction Coefficient Set

**Figure 15:**
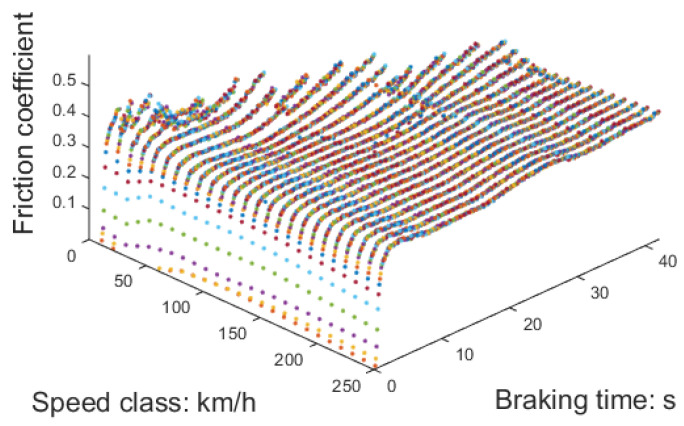
Friction coefficient set generated by LSTM.

By utilizing the friction coefficient from the test as the training set, LSTM is employed to predict the braking duration and the friction coefficient of the brake pad at various other braking speed classes, as shown in Figure 15. The assessment criteria for these predictions include the duration of the braking process and the observed patterns in the friction coefficient.

Given that the response delay of pneumatic components is approximately 50 ms, a time interval of 100 ms was selected. Moreover, a speed class interval of 10 km/h was selected, and the subsequent test results validated that this data scale meets the requirements of the braking system. The calculation workload is not excessively high, ensuring that it does not impact the braking response time, and the data intervals are not too sparse to compromise accuracy.

### 6.4. Line Test

The test was conducted on a specific three-kilometer route that includes ramps and has a maximum gradient of 5‰, with a speed of 100 km/h. Different braking conditions were examined. The test vehicle was set up to mimic the typical operating load of AW2.

**Figure 16:**
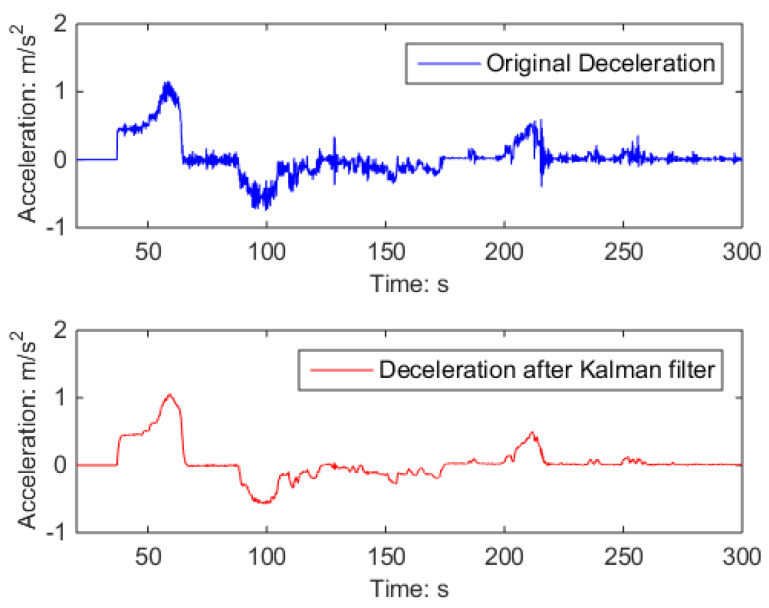
Kalman filtering effect.

The signals collected by the deceleration sensor indicate that the application of Kalman filtering and digital signal processing techniques successfully mitigated noise and the acceleration–deceleration oscillations resulting from uneven speed during vehicle operation, as depicted in Figure 16. Nevertheless, the variations in deceleration throughout the braking process were still detected and maintained.

**Figure 17:**
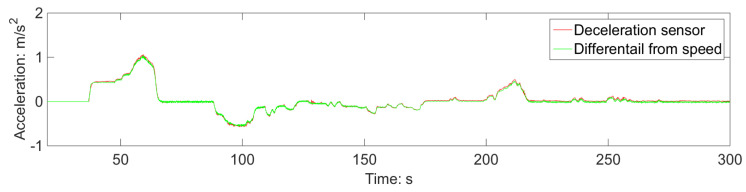
Impact of rampway angle on deceleration measurement.

Since the test route does not include any curves, it was not possible to assess the road conditions related to curves using acceleration information. However, Figure 17 provides valuable insights, indicating a slight disparity of 0.005 m/s^2^ between the deceleration sensor signal and the signal derived from the speed differential solution. This suggests a nominal rampway angle of approximately 5‰.

**Figure 18:**
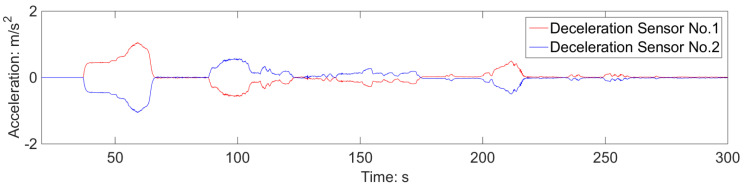
Deceleration signals of the first and last trains.

Furthermore, upon comparing the deceleration signals of the first and last carriage, it can be observed that the deceleration of the entire train is consistent, as depicted in Figure 18. This can be ascribed to the confined working conditions of the train, characterized by swift transitions from acceleration to deceleration, treating the train as a cohesive entity. However, it should be noted that this scenario may not hold true for long-term operating conditions.

**Figure 19:**
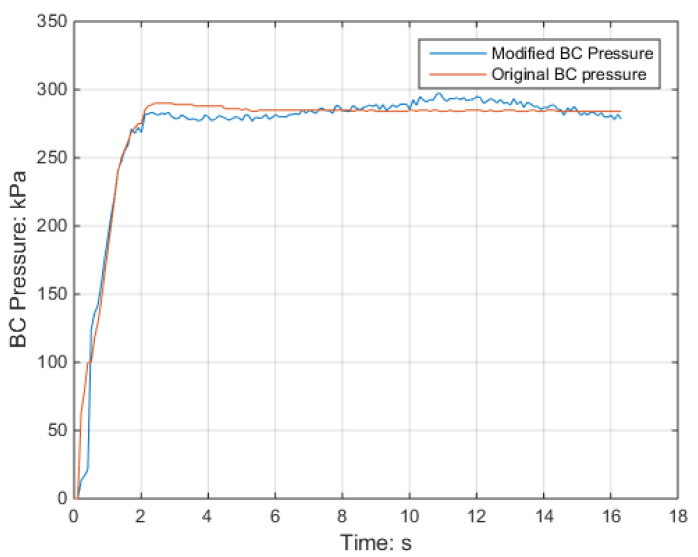
Brake cylinder pressure before and after modification.

**Figure 20:**
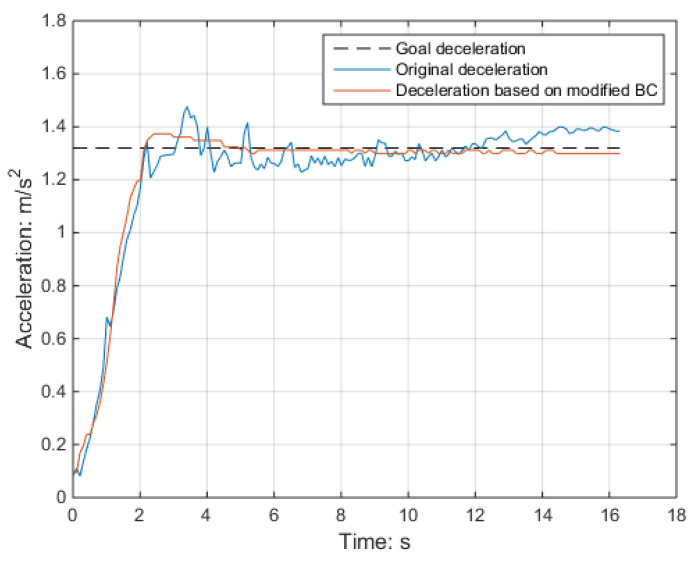
Brake cylinder pressure before and after modification.

The brake cylinder pressure and deceleration during the braking process were analyzed for the selected speed classes, with a focus on a lower speed of 60 km/h for safety purposes. Figure 19 and Figure 20 display the brake cylinder pressure and deceleration data, respectively, for both the original and modified methods. The brake cylinder pressure was obtained using a pressure sensor in the BCU, while the deceleration was measured using a deceleration sensor. It is evident from the results that the modified method leads to smoother and more closely aligned deceleration with the target deceleration of 1.32 m/s^2^. This demonstrates the effectiveness of adjusting the brake cylinder pressure using the dynamic friction curve.

## 7. Discussion

The aforementioned prototype of the deceleration feedback braking control technology demonstrates the achievement of smooth deceleration through experimental validation. However, there are several areas that offer the potential for improvement:

The weight distribution among different carriages during actual operation may not be as optimal as in the test train due to elastic connections. This can lead to inconsistent deceleration among the carriages and the entire train. To improve precise deceleration control, the sensor arrangement can be further optimized, for instance by using sensors for each trailer

In the future, there may be faster and more efficient methods to obtain the required set of friction coefficients. Currently, testing dedicated coefficient sets for different brake pads is labor-intensive. Furthermore, a universal coefficient set has not yet been identified, as the temperature rise curve and friction coefficient are significantly influenced by the materials and thermal conductivity of different brake pads.

While the current approach effectively addresses the instability caused by the friction coefficient, it simplifies the adhesion on the track surface. Significant changes in track conditions, such as slippery surfaces, can render the parameter set collected under ideal working conditions ineffective. To address this, separate considerations of dry and wet rail conditions and the further optimization of the coefficient set based on working conditions are being explored.

The application of this technology currently assumes that wheel and rail wear is not severe in the short term. Nevertheless, with the accumulation of operating mileage, the efficiency of braking can be affected by wheel and rail wear, causing changes in the proportional relationship between the original brake cylinder pressure and deceleration. The design team has considered employing a dynamic simulation algorithm that automatically updates to adapt the coefficient set to the degradation process. However, distinguishing long-term decay from short-term deterioration caused by humidity is challenging within short time frames.

## 8. Conclusions

This research introduces a deceleration feedback-based braking system with a closed-loop control function. The system aims to tackle challenges associated with the fluctuating friction coefficient between the brake disc and pad when braking. By automatically adapting the brake cylinder pressure, the system maintains a stable actual deceleration at the target value, thus achieving the desired braking distance. To ensure accuracy in monitoring train deceleration, a specialized deceleration sensor is applied. Machine learning techniques are utilized to determine the friction coefficient based on braking time, temperature, and different speed classes, using data from brake disc and pad tests. This approach effectively mitigates oscillation caused by fluctuations in the friction coefficient during braking. Experimental tests are conducted on a test line to evaluate the system’s performance. The test results demonstrated that the technique was effective in achieving a more precise alignment between the actual deceleration and the target deceleration, while also preventing wheel sliding protection (WSP) activation, particularly during low-speed periods. Furthermore, the implementation of this technique did not cause any delays or disruptions to the braking time or the BCU operation.

In the future, this work aims to integrate deceleration feedback information from all trains on the same line and develop an automated platform for updating the dynamic friction coefficient set. The revised friction coefficient set will consider factors such as the wheel–rail adhesion coefficient and environmental humidity.

## Figures and Tables

**Table 1:** Parameters of deceleration sensors.

Parameters	Value	Units
Dynamic range	±2	g
Zero acceleration output	±15	mV
Sensitivity	1000	mV/g
Noise density	550	µV RMS
Frequency response –3 dB	0–94	Hz
Shock limit	5000	g
Transverse sensitivity	<3	%
Weight	38	Grams

## Data Availability

Not applicable.
